# Attached compared with unattached surface probes for monitoring flap perfusion in microvascular head and neck reconstruction: a feasibility study

**DOI:** 10.1038/s41598-023-43151-5

**Published:** 2023-09-24

**Authors:** Mark Ooms, Philipp Winnand, Marius Heitzer, Florian Peters, Anna Bock, Marie Sophie Katz, Frank Hölzle, Ali Modabber

**Affiliations:** Department of Oral and Maxillofacial Surgery, University Hospital RWTH Aachen, Pauwelsstraße 30, 52074 Aachen, Germany

**Keywords:** Medical research, Diagnostic markers

## Abstract

Unattached surface probes are commonly used with the O2C analysis system (LEA Medizintechnik, Germany) to monitor microvascular free flap perfusion. This study compared attached and unattached surface probes for extraoral free flaps. The study included 34 patients who underwent extraoral microvascular head and neck reconstruction between 2020 and 2022. Flap perfusion was monitored postoperatively using the O2C analysis system at 0, 12, 24, 36, and 48 h, with an attached surface probe at 3 mm tissue depth and an unattached surface probe at 2 mm and 8 mm tissue depths. Clinical complications, technical errors, and perfusion measurement values were compared. No clinical complications (attachment suture infections) or technical errors (probe detachment) occurred. Flap blood flow values of the probes were partially different (3 mm vs. 2 and 8 mm: p < 0.001; p = 0.308) and moderately correlated (3 mm with 2 and 8 mm: r = 0.670, p < 0.001; r = 0.638, p < 0.001). Hemoglobin concentration and oxygen saturation values were generally different (3 mm vs. 2 and 8 mm: all p < 0.001) and variably correlated (3 mm with 2 and 8 mm: r = 0.756, r = 0.645; r = 0.633, r = 0.307; all p < 0.001). Both probes are comparable in terms of technical feasibility and patient safety, with flap perfusion values dependent on tissue measurement depth.

## Introduction

The use of microvascular free flaps is a reliable and efficient method for head and neck reconstruction offering advantages such as the ability to repair complex structures and a high overall success rate of over 95% for all flaps in general^1–3^. However, flap failures may still occur and have devastating consequences for the patient, such as the need for additional surgical procedures and potentially permanent disfigurement and functional impairment^3,4^.

Since anastomotic compromise occurs in approximately 10% of all cases and timely recognition and intervention are the most important factors for successful flap salvage, regular postoperative assessment of flap viability is crucial for microvascular free tissue transfer^1,5–8^. The standard clinical assessment of flap viability, which considers parameters such as flap surface color, temperature, and capillary refill, faces complications due to delayed external signs of impaired flap perfusion and the natural paleness of flaps, and is also subjective in nature, requiring experienced personnel who may not always be available^1,4,9,10^. Therefore, technical methods for flap monitoring, such as the oxygen-to-see (O2C) analysis system (LEA Medizintechnik, Germany), have been developed^1,11^.

The O2C analysis system uses unattached surface probes to measure flap perfusion at tissue depths of 2 mm and 8 mm^6,11,12^. It is an established and reliable method for assessing flap perfusion as a prerequisite for flap viability and meets several of the requirements for an ideal flap monitoring method, such as objectivity, accuracy, and the ability to distinguish between arterial and venous congestion^1,6,11–13^. A lighter, smaller, and, as a result, attachable surface probe that measures flap perfusion at a 3 mm tissue depth has recently been developed, with the potential advantages of no changes in probe contact pressure or measurement location over the postoperative monitoring period, both of which may affect flap perfusion measurement^14–16^. Nevertheless, damages to the microvasculature of the flap, infection and bleeding due to attachment sutures, or technical errors due to probe detachment or disconnection are potential disadvantages^17^.

The aim of this study was to evaluate the use of attached surface probes with the O2C analysis system for monitoring flap perfusion of any flap that has a skin island and to compare attached surface probes and standard unattached surface probes in terms of technical feasibility, patient safety and perfusion measurement values.

## Results

### Study population

The study population consisted of 34 patients, 19 men and 15 women (Table 1). The flap type for reconstruction was RFFF in 14 patients, ALTF in 12 patients, and FFF in 8 patients.

**Table 1:** Clinical characteristics of the study population.

Number	34
Sex (n)	
Male	19 (55.9%)
Female	15 (44.1%)
Age (years)	70.5 (20.0)
BMI (kg/m^2^)	24.1 (5.9)
ASA (n)	
1	0 (0.0%)
2	13 (38.2%)
3	19 (55.9%)
4	2 (5.9%)
Flap type (n)	
RFFF	14 (41.2%)
ALTF	12 (35.3%)
FFF	8 (23.5%)
Flap location (n)	
Submandibular	12 (35.3%)
Cheek	5 (14.7%)
Infraorbital	2 (5.9%)
Nasal	2 (5.9%)
Orbital	5 (14.7%)
Auricular	6 (17.6%)
Temporoparietal	2 (5.9%)
Arterial anastomosis recipient vessel (n)	
External carotid artery	9 (26.5%)
Facial artery	17 (50.0%)
Superior thyroid artery	8 (23.5%)
Venous anastomosis recipient vessel (n)	
Internal jugular vein	19 (55.9%)
Internal jugular vein + other vein	6 (17.6%)
Other vein	9 (26.5%)
Surgery duration (min)	496.0 (144.0)
Flap ischemia duration (min)	99.0 (28.0)

Parameters are indicated as numbers (with percentage) for categorical data (sex, ASA, flap type, flap location, arterial anastomosis recipient vessel, venous anastomosis recipient vessel) or median (with interquartile range) for metric data (age, BMI, surgery duration, flap ischemia duration).

*BMI* body mass index, *ASA* American Society of Anesthesiologists score, *RFFF* radial free forearm flap, *ALTF* anterolateral thigh flap, *FFF* fibular free flap.

### Clinical complications and technical errors

No clinical complications, such as flap failure or the need for flap revision, were observed. Furthermore, no infection or bleeding due to the attachment sutures and no technical errors such as probe detachment or disconnection were observed with the attached surface probe.

### Flap perfusion measurement values

**Figure 1:**
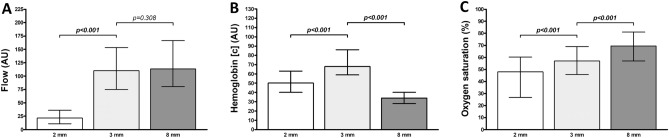
Differences between perfusion measurement values. Data shown as median (with interquartile range) for blood flow (**A**), hemoglobin concentration (**B**), and oxygen saturation (**C**) at 2 mm, 3 mm, and 8 mm tissue measurement depths for all measurement timepoints combined; p-values corresponding to testing for differences between tissue measurement depths with Wilcoxon signed-rank test (3 mm vs. 2 mm and 3 mm vs. 8mm); significant p-values are bold. *AU* arbitrary units.

The blood flow values measured at 3 mm and 2 mm tissue depths differed, but no difference between the values at 3 mm and 8 mm tissue depths were seen when considering all measurement time points separately and combined (all p < 0.05 and all p > 0.05, respectively) (Table 2, Fig. 1).

**Table 2:** Comparison of flap perfusion measurement values.

Tissue depth	Timepoint of measurement					All
	0 h	12 h	24 h	36 h	48 h	
Blood flow (AU)						
2 mm	20.5 (24.0)	24.0 (28.0)	17.5 (23.0)	26.0 (24.0)	26.0 (28.0)	21.5 (25.0)
3 mm	99.5 (74.0)	103.0 (78.0)	112.5 (93.0)	116.5 (71.0)	124.5 (68.0)	110.0 (79.0)
8 mm	92.0 (67.0)	105.5 (62.0)	125.0 (82.0)	125.5 (103.0)	139.0 (105.0)	113.5 (86.0)
p-value 1	** < 0.001**	** < 0.001**	** < 0.001**	** < 0.001**	** < 0.001**	** < 0.001**
p-value 2	0.936	0.824	0.897	0.578	0.274	0.308
Hemoglobin concentration (AU)						
2 mm	58.0 (26.0)	53.0 (24.0)	49.5 (20.0)	48.5 (18.0)	44.0 (23.0)	50.0 (23.0)
3 mm	67.5 (31.0)	69.0 (29.0)	68.5 (27.0)	67.0 (26.0)	68.5 (26.0)	68.0 (27.0)
8 mm	33.0 (10.0)	32.0 (12.0)	35.0 (12.0)	33.5 (13.0)	36.0 (14.0)	34.0 (12.0)
p-value 1	** < 0.001**	** < 0.001**	** < 0.001**	** < 0.001**	** < 0.001**	** < 0.001**
p-value 2	** < 0.001**	** < 0.001**	** < 0.001**	** < 0.001**	** < 0.001**	** < 0.001**
Hemoglobin oxygen saturation (%)						
2 mm	51.0 (36.0)	43.0 (31.0)	50.0 (37.0)	46.0 (31.0)	46.5 (40.0)	48.0 (34.0)
3 mm	61.0 (24.0)	47.5 (32.0)	56.5 (21.0)	58.0 (22.0)	62.0 (24.0)	57.0 (23.0)
8 mm	69.5 (27.0)	69.5 (30.0)	67.0 (25.0)	70.0 (25.0)	70.0 (19.0)	69.5 (24.0)
p-value 1	** < 0.001**	**0.006**	0.056	**0.001**	** < 0.001**	** < 0.001**
p-value 2	**0.005**	** < 0.001**	**0.003**	**0.001**	**0.029**	** < 0.001**

Perfusion measurement values are indicated as median (with interquartile range) for each measurement time point (0 h postoperatively, 12 h postoperatively, 24 h postoperatively, 36 h postoperatively, 48 h postoperatively) and for all measurement time points combined (all); p-values corresponding to testing for differences between tissue measurement depths with Wilcoxon signed-rank test (p-value 1: 3 mm vs. 2mm; p-value 2: 3 mm vs. 8mm); significant p-values are bold.

*AU* arbitrary units.

Differences were observed between the hemoglobin concentration values at 3 mm and 2 mm tissue depths and between those at 3 mm and 8 mm tissue depths when considering all measurement time points separately and combined (all p < 0.05) (Table 2, Fig. 1).

Furthermore, differences between the hemoglobin oxygen saturation values at 3 mm and 2 mm tissue depths were seen for the measurement time points at 0, 12, 36, and 48 postoperative hours and for all measurement time points combined (all p < 0.05). The values measured 24 h postoperatively did not differ (p = 0.056) (Table 2, Fig. 1). The Hemoglobin oxygen saturation values at 3 mm and 8 mm tissue depths differed for all measurement time points separately and combined (all p < 0.05) (Table 2, Fig. 1).

Analysis of the differences between the perfusion measurements, separately for each flap type, showed similar results for blood flow and hemoglobin concentration for all flaps, while results for hemoglobin oxygen saturation were similar only for the ALTF (Table 3).

**Table 3:** Comparison of flap perfusion measurement values separately for flap type.

Tissue depth	Timepoint of measurement					All
	0 h	12 h	24 h	36 h	48 h	
Blood flow (AU)						
RFFF						
2 mm	37.0 (35.0)*	31.5 (21.0)*	32.5 (41.0)*	27.5 (18.0)*	39.0 (24.0)*	32.5 (26.0)*
3 mm	124.5 (134.0)	145.5 (121.0)	149.0 (125.0)	157.0 (129.0)	148.0 (124.0)	140.5 (128.0)
8 mm	102.5 (95.0)	110.0 (55.0)	131.0 (58.0)	122.0 (110.0)	159.0 (89.0)	124.5 (81.0)
ALTF						
2 mm	15.0 (14.0)*	12.0 (9.0)*	16.0 (7.0)*	16.5 (32.0)*	16.0 (16.0)*	16.0 (13.0)*
3 mm	87.0 (63.0)	80.0 (33.0)	82.0 (80.0)	101.5 (73.0)	108.5 (76.0)	91.5 (53.0)
8 mm	73.0 (34.0)	83.0 (31.0)	102.0 (93.0)	107.0 (129.0)	116.0 (115.0)	84.0 (88.0)
FFF						
2 mm	19.5 (19.0)*	25.0 (45.0)*	15.5 (24.0)*	28.5 (37.0)*	27.0 (26.0)*	21.0 (25.0)*
3 mm	92.5 (97.0)	107.0 (65.0)	94.0 (65.0)	109.0 (44.0)	112.5 (36.0)	106.5 (54.0)
8 mm	101.5 (125.0)	126.5 (79.0)	122.5 (99.0)	145.0 (98.0)	165.5 (106.0)	136.5 (90.0)*
Hemoglobin concentration (AU)						
RFFF						
2 mm	69.0 (15.0)*	56.5 (19.0)*	60.0 (21.0)*	55.0 (16.0)*	54.5 (24.0)*	58.0 (21.0)*
3 mm	91.0 (28.0)	82.5 (23.0)	82.5 (22.0)	85.5 (24.0)	85.5 (24.0)	86.5 (24.0)
8 mm	38.5 (20.0)*	35.0 (14.0)*	39.0 (13.0)*	37.0 (14.0)*	40.5 (13.0)*	38.0 (13.0)*
ALTF						
2 mm	43.0 (12.0)*	43.0 (15.0)*	42.0 (9.0)*	42.5 (15.0)*	38.5 (16.0)*	42.0 (13.0)*
3 mm	63.5 (13.0)	55.5 (15.0)	59.5 (14.0)	59.5 (13.0)	60.5 (12.0)	60.0 (12.0)
8 mm	30.0 (5.0)*	29.0 (6.0)*	29.0 (5.0)*	28.5 (15.0)*	31.0 (12.0)*	30.0 (7.0)*
FFF						
2 mm	60.5 (14.0)*	58.5 (28.0)*	51.0 (17.0)*	50.5 (19.0)*	44.0 (22.0)*	51.5 (22.0)*
3 mm	67.5 (29.0)	70.0 (28.0)	67.0 (26.0)	66.5 (24.0)	67.0 (27.0)	67.5 (22.0)
8 mm	34.5 (5.0)*	37.5 (20.0)*	35.0 (13.0)*	32.0 (11.0)*	36.0 (11.0)*	35.0 (10.0)*
Hemoglobin oxygen saturation (%)						
RFFF						
2 mm	62.5 (22.0)	54.0 (26.0)	61.0 (30.0)	56.5 (32.0)*	54.5 (25.0)*	58.0 (21.0)*
3 mm	68.0 (22.0)	67.5 (31.0)	65.0 (21.0)	69.5 (20.0)	70.5 (13.0)	67.5 (19.0)
8 mm	74.0 (28.0)	69.5 (35.0)	70.5 (29.0)	74.0 (33.0)	67.5 (19.0)	71.0 (28.0)
ALTF						
2 mm	33.5 (29.0)*	27.5 (18.0)*	33.0 (31.0)*	24.5 (34.0)*	13.0 (34.0)*	28.0 (31.0)*
3 mm	48.0 (24.0)	40.0 (13.0)	47.0 (24.0)	48.0 (22.0)	47.0 (24.0)	47.0 (18.0)
8 mm	71.0 (26.0)*	69.0 (25.0)*	69.5 (22.0)*	69.5 (23.0)*	69.5 (29.0)*	69.5 (23.0)*
FFF						
2 mm	47.0 (49.0)	48.0 (36.0)	52.5 (62.0)	46.0 (24.0)	46.5 (38.0)	47.5 (37.0)*
3 mm	57.0 (23.0)	51.0 (36.0)	51.0 (33.0)	52.0 (23.0)	54.0 (26.0)	54.0 (25.0)
8 mm	67.5 (24.0)	70.5 (25.0)	64.5 (25.0)	69.5 (26.0)	71.5 (24.0)	68.5 (21.0)*

Perfusion measurement values are indicated as median (with interquartile range) for each measurement time point (0 h postoperatively, 12 h postoperatively, 24 h postoperatively, 36 h postoperatively, 48 h postoperatively) and for all measurement time points combined (all) separately for flap type (RFFF, ALTF and FFF); *p < 0.05 corresponding to testing for differences between tissue measurement depths with Wilcoxon signed-rank test (2 mm or 8 mm vs. 3 mm).

*AU* arbitrary units, *RFFF* radial free forearm flap, *ALTF* anterolateral thigh flap, *FFF* fibula free flap.

**Figure 2:**
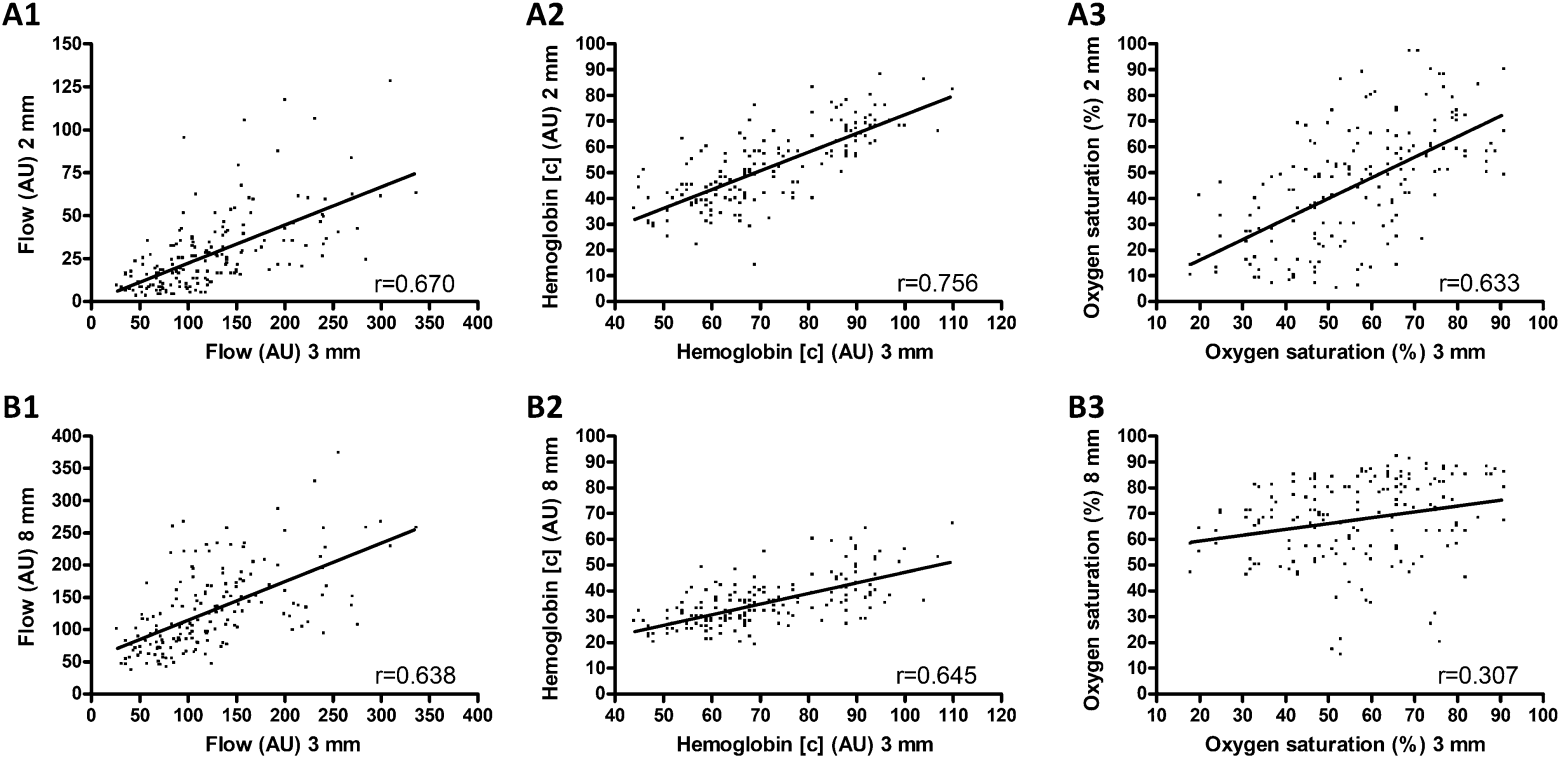
Associations between perfusion measurement values. Data shown as scatter plot for blood flow (l), hemoglobin concentration (2), and hemoglobin oxygen saturation (3) at 2 and 3 mm tissue depths (**A**) and at 8 and 3 mm tissue depths (**B**); r = Spearman correlation coefficient; trend lines are corresponding to linear regression analysis (blood flow at 2 and 3 mm tissue depths: 0.21 + 0.22x; hemoglobin concentration at 2 and 3 mm tissue depths: -0.17 + 0.72x; hemoglobin oxygen saturation at 2 and 3 mm tissue depths: 0.19 + 0.79x; blood flow at 8 and 3 mm tissue depths: 54.83 + 0.59x; hemoglobin concentration at 8 and 3 mm tissue depths: 6.04 + 0.41x; hemoglobin oxygen saturation at 8 and 3 mm tissue depths: 54.78 + 0.22x). *AU* arbitrary units, [c] = concentration.

The results showed a moderate correlation between the 3 mm and 2 mm tissue depth measurements of blood flow and hemoglobin oxygen saturation and a strong correlation between the values of hemoglobin concentration (r = 0.670, r = 0.633, r = 0.756, respectively; all p < 0.001) (Fig. 2). Moderate correlations were found between the 3 mm and 8 mm tissue depth measurements of blood flow and hemoglobin concentration, while the hemoglobin oxygen saturation values were weakly correlated (r = 0.638; r = 0.645; r = 0.307, respectively; all p < 0.001) (Fig. 2).

## Discussion

The postoperative monitoring of flap viability in microvascular free flap reconstruction is essential for the timely correction of any vascular compromise, and the use of unattached surface probes with the O2C analysis system has been shown to be a reliable method for monitoring flap perfusion as a prerequisite for flap viability^1,6,7,11^. Attached surface probes have been developed for the O2C analysis system because of their advantages, such as limited interference from changes in probe pressure and the location of the measurement area, and they provide the basis for continuous flap perfusion monitoring over 24 h a day^14–16^. Nevertheless, flap failure due to damage to the flap microvasculature, infection and bleeding due to probe fixation sutures, and technical errors due to probe detachment or disconnection are the potential disadvantages of attached surface probes^17^. Therefore, in the present study, attached surface probes were compared with unattached surface probes in terms of technical feasibility, patient safety, and perfusion measurement values^6,11,12,18^. To reduce the influence of the moist environment during measurement, which is generally more pronounced for intraoral flaps, only extraoral flaps were investigated^19^.

This study demonstrated that using attached surface probes with the O2C analysis system does not compromise technical feasibility or patient safety. No probe detachment or disconnection was observed with the attached surface probes, which could be attributed to the sufficient fixation of the probe with four sutures on the cutaneous flap surface and to the additional securing of the probe connection cable with patches on the skin beyond the flap. No damage to the microvasculature of the flap, which could lead to local or general flap necrosis, was observed. A possible explanation for this is that the sutures were superficial and not too close to the intraoperatively identified perforator vessel of the ALTF or FFF; both perforator flaps that depend on only one perforator vessel or a limited number of perforator vessels^20^. No infections or bleeding were observed upon using the attached surface probes, likely because of adherence to sterile conditions during fixation and the superficial design of the attachment sutures.

The findings of this study showed that the attached and unattached surface probes used with the O2C analysis system differed in their quantitative measurements of flap perfusion: differences were seen between the blood flow values at 3 mm and 2 mm tissue depths, the hemoglobin concentration values at 3 mm and 2 mm and 3 mm and 8 mm tissue depths, and hemoglobin oxygen saturation values at 3 mm and 2 mm and 3 mm and 8 mm tissue depths. It should be noted that the attached and unattached surface probes measured flap perfusion at different tissue depths, i.e., 2 mm, 3 mm, and 8 mm. The attached surface probe measures only at one tissue depth, namely 3 mm, to meet the requirements for probe attachment (i.e., light weight and small size). Therefore, considering the anatomical composition of the skin, the differences in the flap perfusion measurement values may be related to variability in the skin microvasculature depending on the skin depth or layer evaluated, that is, the dermis with the superficial papillary and the deeper reticular plexus or the subcutaneous tissue with the subcutaneous plexus^21,22^. For instance, the blood flow and hemoglobin concentration values measured at a 3 mm tissue depth were higher than those measured at 2 mm, which could be due to the more prominent representation of the reticular plexus and its high vessel density at a tissue depth of 3 mm^21,22^. Further, hemoglobin oxygen saturation was higher when measured at a 3 mm tissue depth than at 2 mm, which may be explained by the dependence of hemoglobin oxygen saturation on blood flow^11^. However, variations in flap perfusion due to the narrow but non identical measurement areas of the attached and unattached surface probes cannot be excluded^14,15^. Notably, hemoglobin oxygen saturation being lower at the 3 mm tissue depth than at 8 mm, despite similarity in the blood flow, may reflect higher oxygen extraction in the dermal layer than in the subcutaneous tissue, because the measurement of hemoglobin oxygen saturation methodically reflects the oxygen saturation level of hemoglobin in the capillary venous vasculature after oxygen extraction^11^. In addition, the flap perfusion measurements at tissue depths of 3 mm, 2 mm, and 8 mm were differently correlated. For example, the 3 mm and 2 mm measurements of hemoglobin concentration were strongly correlated, whereas the 3 mm and 8 mm measurements of hemoglobin oxygen saturation were weakly correlated. The observed differences between and variable correlations among the measured flap perfusion values imply that cut-off values indicating sufficient flap perfusion for unattached surface probe measurements at a 3 mm tissue depth cannot be directly derived from previously determined cut-off values for unattached surface probe measurements at 2 mm and 8 mm tissue depths^6,11^.

The small sample of patients and the lack of failed flaps or flaps requiring revision because of vascular compromise, which made it impossible to evaluate cut-off values for unattached surface probe measurements at a 3 mm tissue depth, were the limitations of the present study. However, this was a pilot study that evaluated the use of attached surface probes with the O2C analysis system for flap perfusion monitoring for the first time, and generally only few flaps require revision in microvascular head and neck reconstruction^1,4,23^. In addition, the short 48-h time interval for the comparison of attached and unattached surface probes in this study was based on the observation that flap monitoring is most valuable in the first 48 postoperative hours, because of the high risk of vascular comprise during this period and the low salvage rates for failing flaps beyond this period^8,24–27^.

This pilot study was conducted to evaluate for the first time the use of attached surface probes with the O2C analysis system, which has already been shown to be a reliable method for flap perfusion monitoring in microvascular head and neck reconstruction with unattached surface probes^6,11,28^. The findings showed that attached surface probes were comparable to commonly used unattached surface probes in terms of technical feasibility and patient safety. Moreover, attached surface probes could also provide the basis for continuous 24-h flap perfusion monitoring as the next step in flap perfusion monitoring. However, in terms of the quantitative measurements and thus the agreement between and interchangeability of attached and unattached surface probes, the attached surface probes studied were not comparable to the commonly used unattached surface probes, and calculation of measurement values for one surface probe based on the measurement values of the other surface probe are not accurate. With regard to clinical implications, further studies are needed to determine cut-off values distinguishing between sufficient and insufficient flap perfusion when using attached surface probes with the O2C analysis system and, consequently, to preserve the usefulness of the O2C analysis system for making decisions about the need for flap revision in microvascular head and neck reconstruction.

## Conclusion

This study showed that in terms of technical feasibility and patient safety, attached surface probes are comparable to the standard unattached surface probes used with the O2C analysis system for flap perfusion monitoring. However, cut-off values for attached surface probes that indicate sufficient flap perfusion need to be determined to maintain the reliability and usefulness of the O2C analysis system as a method for flap perfusion monitoring.

## Methods

### Study population

**Figure 3:**
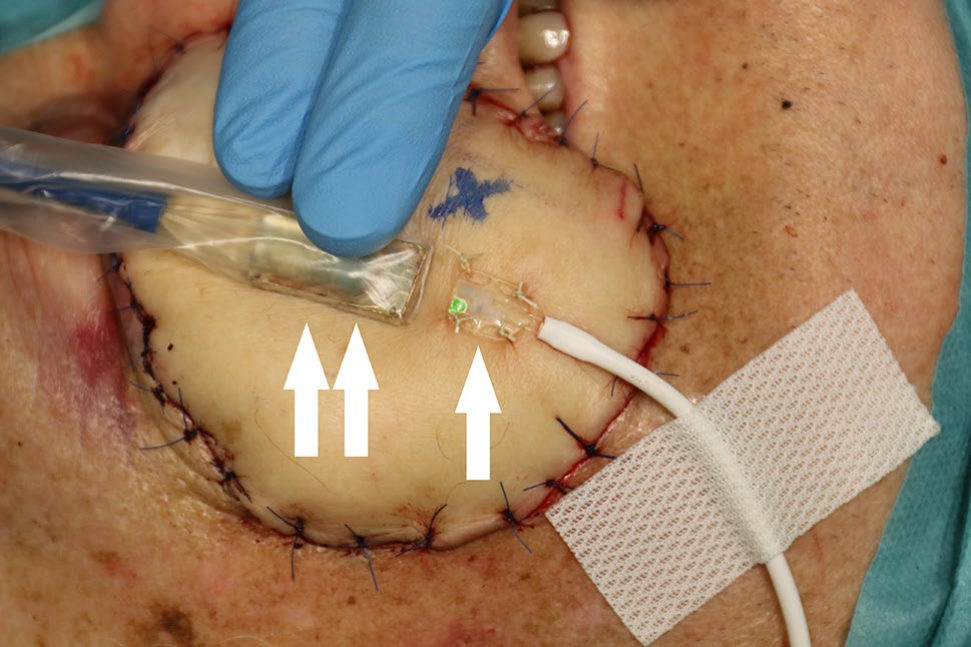
Measurement of flap perfusion. Measurement of perfusion of an ALT flap localized infraorbital right at a 3 mm tissue depth with the attached probe (single arrow) and at 2 and 8 mm tissue depths with the unattached probe (double arrows); cross marking for the perforator.

This study was approved by the local ethics committee of the Medical Faculty RWTH Aachen University (EK 22-358). Informed consent for the use of the study data was waived by the local ethics committee of the Medical Faculty RWTH Aachen University. Informed consent was obtained for publication of the image (Fig. 3) in an online open access publication. All methods were in accordance with the relevant guidelines and regulations. Only patient data without direct involvement of humans were used for the study. All patient data were collected for clinical purposes and retrospectively analyzed.

The study population consisted of 34 patients who had undergone extraoral microvascular head and neck reconstruction with a free tissue flap, i.e., a radial free forearm flap (RFFF), an anterolateral thigh flap (ALTF), or a fibula free flap (FFF), for reconstructive purposes after the resection of a malignant or nonmalignant disease at the Department of Oral and Maxillofacial Surgery between 2020 and 2022. Patients over 18 years of age with complete data records were included. Exclusion criteria were intraoral free flaps.

Data were obtained from the patients’ clinical records. Surgery duration was defined as the time from the first incision to the last suture, and flap ischemia duration was defined as the time from the interruption of flap perfusion at the donor site to the onset of flap perfusion at the recipient site after anastomosis.

All surgical procedures were performed under general anesthesia. All patients were monitored postoperatively in the intensive care unit, with invasive mechanical ventilation and analgosedation until at least the next morning.

### Flap perfusion measurement data

Flap perfusion measurement was performed postoperatively using the O2C analysis system (Oxygen-to-see O2C, LEA Medizintechnik, Giesen, Germany) at 0, 12, 24, 36, and 48 h, with an unattached surface probe (type LF2) at 2 mm and 8 mm tissue depths and an attached surface probe (type LFX90) at a 3 mm tissue depth (Fig. 3). The attached probe was fixed with four sutures in the middle of the flap for RFFFs and with a distance of 1 cm longitudinally parallel to the perforator vessel for ALTFs and FFFs. The unattached probe was held in the same position parallel to the attached surface probe for each measurement.

The O2C analysis system uses Doppler spectroscopy (830 nm; 30 mW) to determine flap blood flow (arbitrary units [AU]) and white light spectroscopy (500–800 nm; 50 W) to determine hemoglobin concentration (AU) and hemoglobin oxygen saturation (%)^11,18^. The measurement time was 10 s for both probes.

### Statistical analysis

Data were expressed as numbers (with percentages) or median values (with interquartile ranges). Differences between the perfusion measurement values, namely flap blood flow, hemoglobin concentration, and hemoglobin oxygen saturation were analyzed using the Wilcoxon signed-rank test (3 mm vs. 2 mm; 3 mm vs. 8 mm). Associations between the perfusion measurement values, namely flap blood flow, hemoglobin concentration, and hemoglobin oxygen saturation were analyzed by calculating the Spearman correlation coefficient, and trend lines for the corresponding scatter plots were obtained by conducting a linear regression analysis (3 mm and 2 mm; 3 mm and 8 mm). P < 0.05 was considered statistically significant. The statistical analysis was performed using SPSS version 28 (SPSS, IBM, New York, USA).

## Data Availability

The datasets generated and analyzed during the current study are not publicly available due to further analysis but are available from the corresponding author on reasonable request.
